# miR-142-5p as a CXCR4-Targeted MicroRNA Attenuates SDF-1-Induced Chondrocyte Apoptosis and Cartilage Degradation via Inactivating MAPK Signaling Pathway

**DOI:** 10.1155/2020/4508108

**Published:** 2020-01-24

**Authors:** Yaoyv Xiang, Yanlin Li, Lingjian Yang, Yinghong He, Di Jia, Xidan Hu

**Affiliations:** ^1^Department of Sports Medicine, First Affiliated Hospital of Kunming Medical University, Kunming, Yunnan 650000, China; ^2^Department of Pharmacy, First Affiliated Hospital of Kunming Medical University, Kunming, Yunnan 650000, China

## Abstract

Osteoarthritis (OA) is a chronic joint function disorder with characteristics of chondrocytes reduction and extracellular matrix (ECM) components destruction. MicroRNAs (miRNAs) and the SDF-1/CXCR4 axis are essential factors of chondrocyte apoptosis and ECM degeneration. However, very few studies have investigated the correlation between miRNAs and the SDF-1/CXCR4 axis in osteoarthritis so far. Here, through miRNAs microarray and bioinformatics analyses, we identified miR-142-5p as a CXCR4-targeted and dramatically downregulated miRNA in cartilage from OA patients, as well as in SDF-1-induced OA chondrocytes *in vitro*. In SDF-1-treated primary human OA chondrocytes that were transfected with a miR-142-5p mimic or inhibitor, the expression of CXCR4 was found to be inversely correlated with the expression of miR-142-5p. The dual luciferase reporter assay further verified the target relationship between miR-142-5p and CXCR4. Overexpression of miR-142-5p alleviated OA pathology by suppressing chondrocyte apoptosis, even in CXCR4 overexpressed OA chondrocytes. This was associated with decreased cartilage matrix degradation, reduced cartilage inflammation, and inactivated MAPK signaling pathway. Our study suggests that upregulated expression of CXCR4-targeted miR-142-5p can inhibit apoptosis, inflammation, and matrix catabolism and inactivate the MAPK signaling pathway in OA chondrocytes. Our work provides important insight into targeting miR-142-5p and the SDF-1/CXCR4 axis in OA therapy.

## 1. Introduction

Osteoarthritis (OA) is the most common form of joint function disorders, with the main symptoms including severe pain and joints stiffness, and is also a leading cause of disability in the elderly worldwide [1]. Pathological changes in OA are characterized by synovial inflammation, chondrocytes reduction, and extracellular matrix (ECM) components destruction [2]. Nonetheless, effective therapeutic approaches for OA are restricted to anti-inflammatory drugs, physical therapy for primary symptomatic relief, and knee replacement surgery for late-phase OA patients [3, 4] because of the limited understanding of OA pathogenesis.

Increasing evidence has indicated that the SDF-1/CXCR4 (stromal cell-derived factor-1/CXC motif chemokine receptor type 4) pathway in articular cartilage is closely linked to OA development [5]. CXCR4 is a G protein-coupled receptor that is involved in homing and chemotaxis in the hematopoietic and immune systems, and the ligand of CXCR4 is SDF-1 (also named CXCL12), which is associated with stem cell migration [6]. CXCR4 plays a role in several diseases, including human immunodeficiency virus (HIV) infection, cancer, immunodeficiency, pulmonary artery hypertension (PAH), amyotrophic lateral sclerosis, and pulmonary injury [7]. Recently, accumulating studies have demonstrated that CXCR4 also plays significant roles in OA pathogenesis. For example, CXCR4 expression was identified to be upregulated in the synovial tissues of the OA patients by bioinformatics analyses [8]. In addition, early study demonstrated that a remarkable increased SDF-1 level had been found in the synovial fluid from patients with OA and rheumatoid arthritis [9]. Xu et al. demonstrated that the SDF-1 level was associated with the radiographic severity of OA [10]. Wang et al. indicated that Celastrol treatment attenuated joint pain and cartilage damage in OA rats by suppression of the SDF-1/CXCR4 pathway [11]. Moreover, SDF-1-induced cartilage degradation and Matrix Metalloprotease-3 (MMP-3) and MMP-9 expressions can be attenuated by the administration of CXCR4 antagonists like TN14003 and AMD3100 *in vivo* [12, 13].

Notably, the SDF-1/CXCR4 signaling axis has been unraveled to be able to inhibit aggrecan expression and lessen cartilage degeneration in posttraumatic osteoarthritis rat, which is largely dependent on activations of the typical downstream pathways like NF-*κ*B (nuclear factor kappa-light-chain-enhancer of activated B cells) and MAPKs (mitogen-activated protein kinases) pathways. Thus, targeted inhibition of the SDF-1/CXCR4 axis could be an important strategy to attenuate OA initiation and progression.

MicroRNAs are small, single-stranded noncoding RNAs (approximately 17–24 nucleotides in length) that play a key role in regulating genes expression at the posttranscriptional level by binding to the 3′-untranslated region (3′UTR) of target mRNAs through their seed sequences, which often leads to reduced expression of the target genes [14]. MicroRNAs play crucial roles in various essential biological processes, and their abnormal expression is associated with many kinds of human diseases, including OA [15]. For instance, ectopic expression of miR-204 triggers spontaneous cartilage loss and OA development, whereas miR-204 inhibition results in concomitant recovery of prostaglandins synthesis and suppression of inflammatory senescence-associated secretory phenotype (SASP) factors in experimental OA [16]. It has been demonstrated that miR-15b regulates chondrocytes proliferation and apoptosis by targeting IGF1, IGF1R, and BCL2 from patients with condylar hyperplasia [17] and knockdown of microRNA-203 alleviates LPS-induced injury by targeting MCL-1 in C28/I2 chondrocytes [18]. Recent *in vitro* studies have revealed that miR-449a inhibition promotes cartilage regeneration and prevents progression of osteoarthritis [19]. Since the SDF-1/CXCR4 axis plays a pivotal role in the injury and repair of cartilage, exploring the miRNAs that are involved in modulating the functions of the SDF-1/CXCR4 axis could be very helpful to understand the in-depth pathogenesis of OA. However, there have been very few studies investigating the correlation between miRNAs and the SDF-1/CXCR4 axis in osteoarthritis so far.

In the present study, we aimed to explore whether certain miRNAs with aberrant expression in OA cartilage tissues can also target CXCR4 and figure out the potential mechanisms of the beneficial roles of miRNAs in OA pathogenesis. Since previous studies have revealed that dysfunctions of chondrocytes in articular cartilage are generally considered to be indispensable for OA development [20], we tested the impacts of the overexpression of the CXCR4-targeted miRNA miR-142-5p on *in vitro* cultured SDF-1-induced OA chondrocytes and investigated the underlying molecular mechanisms in attenuating chondrocytes apoptosis and degeneration.

## 2. Materials and Methods

### 2.1. Clinical Specimens

Articular cartilage specimens were collected from 4 female OA patients (62.1 ± 6.8 years old) that received total knee arthroplasty. These patients were diagnosed as OA according to the American College of Rheumatology criteria [21]. Normal cartilage tissues were harvested from 3 trauma patients (2 females and 1 male, 27.4 ± 5.4 years old, without OA or RA history) that underwent the amputation. All surgeries were performed at the First Affiliated Hospital of Kunming Medical University between 2017 and 2018. Written informed consent was obtained from each participant, and all the procedures were approved by the Ethics Committee at the First Affiliated Hospital of Kunming Medical University (Kunming, China).

### 2.2. RNA Extraction and Microarray Analysis

Total RNA was extracted from the cartilage samples from OA patients and healthy individuals using the Qiagen RNeasy Mini kit (Qiagen, Hilden, Germany; cat. No. 74106) according to the manufacturer's instructions. The RNA yield and purity were determined using a NanoDrop ND-1000 spectrophotometer (Nanogen Inc., San Diego, CA, USA) and an Agilent 2100 Bioanalyser (Agilent Technologies, Santa Clara, CA, USA). miRNAs were isolated from total RNA using a miRNA isolation kit (Macherey-Nagel, Düren, Germany), according to the manufacturer's protocol. The expression profiles of miRNAs and mRNAs were determined using an miProfile™ human inflammatory miRNA qPCR Arrays (GC08017K18014P; GeneCopoeia, Inc., Rockville, MD, USA) according to manufacturer's instructions. The expression level of each gene in osteoarthritis group was compared with the control group and the 2^−ΔΔCt^ method was used to calculate the relative expression of miRNAs. The threshold of screening differentially expressed miRNAs was set as a fold change >2.0 and a *p* value <0.05.

### 2.3. CXCR4-Targeted miRNA Prediction and Bioinformatics Analysis

Human CXCR4-targeted miRNAs were predicted using the following databases: TargetScan (http://www.targetscan.org/vert_72/), miRDB (http://mirdb.org/index.html), miRWalk (http://mirwalk.umm.uni-heidelberg.de), miRTarBase (http://mirtarbase.mbc.nctu.edu.tw), and RNAhybrid (https://bibiserv.cebitec.uni-bielefeld.de/rnahybrid). Candidate miRNAs were queried using “CXCR4” as a key word. All prediction results of TargetScan, miRDB, miRWalk, and miRTarBase were selected in the current study. The *p* value was calculated with a unilateral Fisher's exact test and corrected by false discovery rate (FDR). A *p* value <0.05 and an FDR <0.05 were considered as statistically significant.

### 2.4. Chondrocytes Isolation and Culture

All OA articular cartilage samples were harvested from the tibial plateau or femoral condyle, while primary chondrocytes were isolated as previously described [12]. Chondrocytes were maintained in DMEM (Gibco; Thermo Fisher Scientific, Inc., Waltham, MA, USA) containing 5% fetal bovine serum (FBS; Gibco; Thermo Fisher Scientific, Inc.) and 1% penicillin-streptomycin (Fungizone; Gibco; Thermo Fisher Scientific, Inc.). During the culturing period, chondrocytes were incubated in an atmosphere at 37°C with 5% CO_2_, and medium was changed every 2 days.

### 2.5. Reverse Transcription and Quantitative Polymerase Chain Reaction (RT-qPCR)

To mimic the microenvironment in the knee joint of OA patient and healthy individual, primary chondrocytes were divided into the following two groups: the control group and the SDF-1 treatment group. Chondrocytes were treated with 100 ng/ml SDF-1 (PeproTech, Rocky Hill, NJ, USA) for 24 hours. Total RNA was isolated by TRIzol (Invitrogen; Thermo Fisher Scientific, Inc.). After miRNA extraction, reverse transcription was performed with TaqMan MicroRNA Reverse Transcription Kit (Applied Biosystems, Carlsbad, CA, USA) according to the manufacturer's instruction. RNA quantity and quality were determined by a NanoDrop ND-1000 Spectrophotometer (Nanogen Inc., USA). RNA was reverse-transcribed into cDNA by a RevertAid First Strand cDNA Synthesis Kit (K1622, Tiangen Biotech, Beijing, China) and miRcute Plus miRNA First-Strand cDNA Synthesis Kit (KR211-02, Tiangen Biotech, Beijing, China) according to the manufacturer's protocols. The following cycling conditions were used: 45 cycles of denaturation at 94°C for 20 s and amplification at 60°C for 34 s. All reactions were conducted in triplicate and normalized using the miRNA housekeeping gene U6 or mRNA housekeeping gene GAPDH. Expression of relative genes was calculated using the 2^−ΔΔCt^ method [22]. The following primers were used: miR-150-5p, sense, 5′-TCGGCGTCTCCCAACCCTTGTAC-3′, anti-sense, 5′-GTCGTATCCAGTGCAGG GTCCGAGGT-3′; miR-142-5p, sense, 5′-CAUAAAGUAGAAAGCACUACU-3′, anti-sense, 5′-UAGUGCUUUCUACUUUAUGUU-3′; miR-622, sense, 5′-ATCC CAGGGAGACAGAGATCGAGG-3′, anti-sense, 5′-AAGCTTGGTGGTGGACTT TTGGTTGT-3′; miR-513a-5p, sense, 5′-GTCGTATCCAGTGCGTGTCGTGGAGT CGGC-3′, anti-sense, 5′-AATTGCACTGGATACGACATGACA-3′; U6, sense, 5′-T GCGGGTGCTCGCTTCGGCAGC-3′, anti-sense, 5′-CCAGTGCAGGGTCCGAGG T-3′; BAX, sense, 5′-AAGAAGCTGAGCGAGTGT-3′, anti-sense, 5′-GGCGGCA ATCATCCTCTG-3′; matrix metalloproteinase-13 (MMP-13), Forward, 5′-GTGGTGATGAAGATG ATT-3′, Reverse, 5′-TTGTAGGATGGTAGTATGA-3′; Bcl2, Forward, 5′-TGCC TTTGTGGA ACTGTA-3′, Reverse, 5′-GAGCAGAG TCTTCAGAGA-3′; GAPDH, Forward, 5′-AAAGGGTCATCATCTCTG-3′, Reverse, 5′-GCTGTTGTCATACTTC TC-3′.

### 2.6. Immunocytochemistry

After medium was removed, the chondrocytes with indicated treatments were washed 3 times with phosphate-buffered saline (PBS). The cells were blocked with normal goat serum (ZLI-9021, ZsBio, Beijing, China) for 60 min at room temperature. The samples were incubated with the primary anti-CXCR4 antibody (ab124824, Abcam, MA, USA; 1 : 300 dilution) overnight at 4°C. Thereafter, the specimens were treated sequentially with secondary antibody (PV-9000 anti-rabbit antibody, ZsBio, Beijing, China) at 37°C for 30 min. After washing 3 times with PBS, the slides were developed in DAB (3,3′-Diaminobenzidine) chromogen (Invitrogen, Carlsbad, CA, USA) for 5 min. The specimens were then counterstained with hematoxylin (Aladdin, Carlsbad, CA, USA) for 2 min. The images of stained chondrocyte specimens were taken with a microscope (Olympus BX53; Shinjuku, Tokyo, Japan).

### 2.7. Cell Transfection

Chondrocytes were cultured in the 6‐well plate at a density of 1 × 10^5^ cells per well. When the cell confluence reached approximately 60%, chondrocytes were transfected with the indicated miRNA mimic, miRNA inhibitor, or mammalian expression plasmids using Lipofectamine 3000 (Invitrogen) according to the manufacturer's instructions. Cells were divided into the following groups: control (untreated cells), miR-142-5p mimic, miR-NC (negative control miRNA), miR-142-5p inhibitor, and inhibitor NC (negative control inhibitor). In some experiments, cells were transfected with miRNA mimic together with CXCR4-expressing mammalian expression vector or controls as specified. At 24 hours after transfection, cells were cultured with medium supplemented with recombinant SDF-1 (100 ng/ml; PeproTech, Rocky Hill, NJ, USA) for another 24 hours. All chemically synthesized sequences were purchased from RiboBio Co., Ltd. (Guangzhou, China).

### 2.8. Apoptosis Assay

The apoptotic rate of SDF-1-treated chondrocytes was analyzed by Annexin-V/FITC Apoptosis Detection Kit (BD Biosciences, Franklin Lakes, NJ, USA) according to the manufacturer's instructions. Apoptosis was detected with a flow cytometer (CyFlow® Space; Partec GmbH, Germany). Data were analyzed with the FloMax® software (Partec GmbH, Germany).

### 2.9. Western Blot Analysis

Chondrocytes after the indicated treatments were harvested and total protein was extracted from cell lysates. The quantity of protein samples in each specimen was determined by a BCA Kit (Beyotime Biotechnology, Nanjing, China) according to the manufacturer's instructions. Protein samples were separated by SDS-PAGE (sodium dodecyl sulphate-polyacrylamide gel electrophoresis) at 60 V for 25 min and then 90 V for 3 h. The proteins were transferred onto polyvinylidene fluoride (PVDF) (Millipore, Billerica, MA, USA) membranes with a transferring voltage of 120 V for 50 min. The membranes were blocked with 5% bovine serum albumin (BSA) at room temperature for 1 h and then were incubated with primary antibodies, including anti-collagen II (1 : 1000, Abcam, Cambridge, MA, USA, ab34712), anti-aggrecan (1 : 1000, Abcam, USA, ab36861), anti-bax (1 : 2000, Abcam, USA, ab32503), anti-Bcl-2 (1 : 1000, Abcam, USA, ab32124), anti-cleaved caspase-3 (1 : 2000, Abcam, USA, ab2302), anti-cleaved PARP1 (1 : 1000, Abcam, USA, ab32064), anti-P65 (1 : 1000, Abcam, USA, ab16502), anti-p-P65 (1 : 2000, Abcam, USA, ab86299), anti-IKB*α* (1 : 1000, Abcam, USA, ab32518), anti-p-IKB*α* (1 : 10000, Abcam, USA, ab133462), anti-MMP-13 (1 : 1000, Abcam, USA, ab39012), anti-CXCR4 (1 : 100, Abcam, USA, ab16502), anti-p-ERK (1 : 1000, Cell Signaling Technology (CST), Danvers, MA, USA, 4370T), anti-ERK (1 : 1000, CST, USA, 4695T), anti-p-JNK (1 : 5000, Abcam, USA, ab76572) anti-JNK (1 : 2000, Abcam, USA, ab208035), anti-p-p38 (1 : 1000, CST, USA, 4511T), anti-P38 (1 : 1000, CST, USA, 8690T), and anti-GAPDH (1 : 2000, Abmart, Berkeley Heights, NJ, USA, P30008). All the primary antibodies were diluted with 0.5% TBST (Tris-buffered saline, 0.5% Tween 20) buffer and were incubated overnight at 4°C. The membranes were washed and incubated for 1 h at room temperature with horseradish peroxidase- (HRP-) linked anti-rabbit IgG secondary antibody (1 : 2000, CST, USA, 7074) at room temperature for 1 h. Protein bands were visualized by the enhanced chemiluminescence (ECL) Kit (Millipore, Billerica, MA, USA), and band intensity was determined on a Bio-Rad Image Lab 5.0 system. All results were repeated at least three times.

### 2.10. Enzyme-Linked Immunosorbent Assay (ELISA)

After the indicated treatment, culture supernatant was collected from 24-well plates and the concentrations of IL-1*β*, IL-10, and TNF-*α* were, respectively, measured by using human IL-6 ELISA Kit (ab46052; Abcam, USA), human IL-10 ELISA Kit (ab46034; Abcam, USA), and human TNF-*α* ELISA Kit (ab181421; Abcam, USA) according to the manufacturer's instructions.

### 2.11. Immunofluorescence Staining

Immunofluorescence staining was performed to evaluate the expression of collagen II and aggrecan in chondrocytes after specified treatments. The samples were rinsed three times in PBS before fixation using 4% paraformaldehyde, which was followed by permeation using 0.2% TritonX-100 diluted in PBS for 20 min. Then, the cells were blocked with 5% goat serum albumin for 60 min at room temperature. After being rinsed with PBS, the samples were incubated with primary antibody collagen II (1 : 300) or aggrecan (1 : 400) overnight at 4°C. Then, plates were rinsed five times in PBST (PBS with 0.5% Tween 20) and incubated with DyLight® 488-labeled secondary antibody (1 : 1000; KPL, Dallas, TX, United States) for 2 h at 37°C. The samples were counterstained with DAPI (4′,6-diamidino-2-phenylindole; Thermo Fisher Scientific) for 5 min at room temperature. Finally, images were captured by an Olympus microscope (Olympus Corporation, Tokyo, Japan).

### 2.12. Dual‐Luciferase Reporter Gene Assay

The fragment corresponding to the wild-type (WT) or mutant (MUT) CXCR4 3ʹ-UTR containing a potential binding site of miR-142-5p was inserted into a reporter vector of the Dual-Luciferase Reporter Assay System kit (Promega, E1910, Madison, WI, USA). Chondrocytes were seeded in 96-well plates and were cotransfected with the reporter plasmid and a miR-142-5p mimic or negative control (NC) miRNA using Lipofectamine 2000 (Invitrogen, 11668). The luciferase activity was measured via the Dual-Luciferase Reporter Assay System (Promega) after 48 h. The ratio of firefly and Renilla luciferase activities was calculated. The miR-142-5p mimic, NC-miRNA, and DNA fragments of CXCR4 3ʹ-UTR-WT and CXCR4 3ʹ-UTR-MUT were purchased from Ruibo Biotechnology Co., Ltd. (Guangzhou, China).

### 2.13. Statistical Analysis

Data were analyzed using SPSS 17.0 statistical software (IBM, Armonk, NY, USA). The results are presented as means ± standard deviation (SD) or means ± standard error of mean (SEM) as specified. Analysis of two groups was achieved using a Student's *t*-test. The statistical significance of variances from multiple groups was calculated using analysis of variance (ANOVA). *p* < 0.05 was considered statistically significant.

## 3. Results

### 3.1. miRNA Microarray and Bioinformatics Analyses Identified That miR-142-5p Expression is Downregulated in Chondrocytes of OA Patients and SDF-1-Treated Chondrocytes

**Figure 1:**
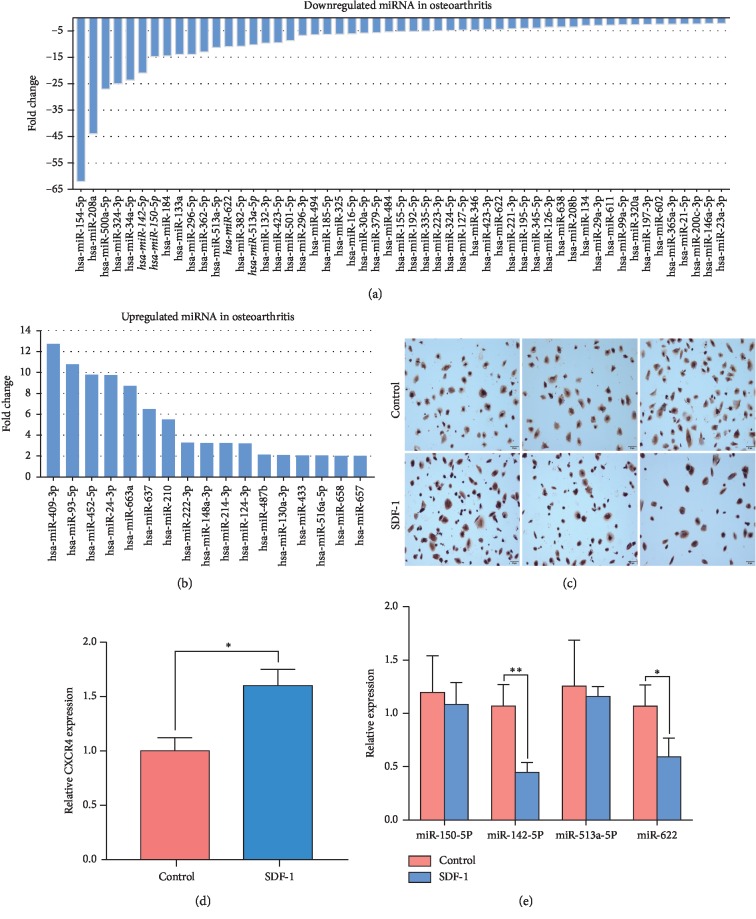
Screening and identification of candidate miRNAs involved in the SDF-1/CXCR4 axis in OA pathogenesis through miRNA microarray and bioinformatics analyses (a, b). The aberrantly expressed miRNAs were screened out by miRNA microarray analyses of cartilage tissue samples from OA patients and healthy cartilage tissue samples. Top downregulated miRNAs (a) and upregulated miRNAs (b) in OA cartilage tissues are shown. (c) CXCR4 expression in SDF-1-induced human chondrocytes by immunohistochemistry staining. Scale bar, 50 *μ*m. (d) Quantification of relative CXCR4 expression in chondrocytes shown in (c). (e) The levels of four most downregulated and putative CXCR4-targeted miRNAs in control and SDF-1-stimulated OA human chondrocytes (100 ng/ml, for 24 h) were determined by qRT-PCR. *n* = 8 for each group. Significant differences are indicated as ^*∗*^*p* < 0.05 and ^*∗∗*^*p* < 0.01. miRNAs, microRNAs; SDF-1, stromal cell-derived factor 1.

To explore the potential roles of miRNAs in OA pathogenesis, we performed with miRNA microarray analysis of the articular cartilages from OA patients and healthy individuals (*n* = 4 for each group) and sought to find out miRNAs with aberrant expression. Our data indicated that 70 miRNAs had significantly altered expression levels in OA patients in comparison to the healthy controls, and these miRNAs included 53 markedly downregulated miRNAs (Figure 1(a)) and 17 evidently upregulated miRNAs (Figure 1(b)) in the OA cartilages tissues (FC ≥ 2 and *p* value <0.05). In order to identify potential miRNAs that target CXCR4, top 15 upregulated and top 15 downregulated miRNAs among 70 aberrantly expressed miRNAs were selected for further prediction by four databases (TargetScan Human 7.2, miRanda, miRTarBase, and RNAhybrid). This resulted in four putative CXCR4-targeted miRNAs: miR-150-5p, miR-142-5p, miR-513a-5p, and miR-622.

Since previous studies have shown that the content of SDF-1 in the joint fluid of OA patients is much higher than that of normal people [9] and the expression of CXCR4, a receptor of SDF-1, was significantly upregulated in OA chondrocytes by the action of SDF-1 [11], consistent with our immunochemistry staining data (Figures 1(c) and 1(d)), we used SDF-1 to treat OA chondrocytes to simulate the long-term environment of OA chondrocytes in vivo and examined whether the expression of the above-mentioned candidate microRNAs in OA chondrocytes was affected by SDF-1 treatment. As shown in Figure 1(e), the expression of miR-150-5p and miR-513a-5p expression were not significantly different between the control and SDF-1 treated groups (*p*=0.4218 and *p*=0.6868, respectively). Meanwhile both miR-142-5p (*p*=0.0309) and miR-622 (*p*=0.0412) were downregulated upon SDF-1 treatments, and miR-142-5p showed more reduction. These results suggested that the expression of miR-142-5p was significantly decreased under the stimulation of SDF-1, which reduced the inhibitory effect of miR-142-5p on the expression of target protein CXCR4.

### 3.2. CXCR4 Is a Direct Target of miR-142-5p and Is Negatively Regulated by miR-142-5p in Chondrocytes of Articular Cartilages

**Figure 2:**
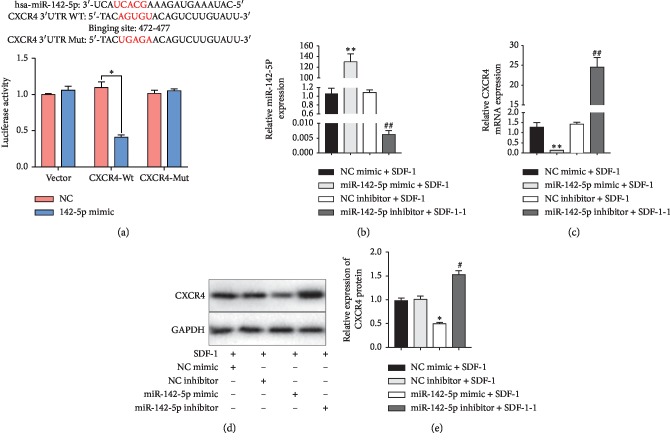
CXCR4 is a direct target of miR-142-5p and is negatively regulated by miR-142-5p. (a) A design scheme showing the binding of miR-142-5p to the predicted 3′UTR site of CXCR4. The binding site of miR-142-5p, AGUGC, was mutated to UGAGC in the reporter vector bearing the mutant 3′UTR. The luciferase activity of the reporter vectors was detected in 293T cells at 48 hours after cotransfection of wild-type (WT) or mutant (Mut) CXCR4 3′UTR with miR-142-5p mimics or negative control mimic. *N* = 3 for each group. (b-e) Primary human OA chondrocytes were transfected with a negative control (NC) mimic, miR-142-5p mimic, NC inhibitor, and miR-142-5p inhibitor and further incubated with SDF-1 (100 ng/ml) for 24 h. The expression levels of miR-142-5p (b) and CXCR4 (c) were then measured by qRT-PCR. The protein levels of CXCR4 were determined by Western blot. The representative bands images are shown (d), and relative bands intensity was summarized (e). *N* = 3 for each group. Data are presented as the mean ± SEM (*n* = 3). ^*∗*^*p* < 0.05; ^*∗∗*^*p* < 0.01*versus* NC mimic; ^#^*p* < 0.05 and ^##^*p* < 0.01*versus* NC inhibitor. miRNAs, microRNAs; SDF-1, stromal cell-derived factor 1; NC, negative control; Gapdh, glyceraldehyde‐3‐phosphate dehydrogenase.

In order to substantiate the binding of miR-142-5p to the target CXCR4, luciferase reporter assays with wild‐type or mutant versions of the 3ʹUTRs of CXCR4 were performed in the presence or absence of miR-142-5p overexpression (Figure 2(a)). The results indicated that miR-142-5p mimic remarkably suppressed the relative luciferase activity of the vector with wild-type CXCR4 3′UTR binding site (*p* < 0.05), whereas mutation of the binding sequences prevented the binding of miR-142-5p and overexpression of negative control mimic resulted in largely unchanged luciferase activity (Figure 2(a)). These results supported the notion that the miR-142-5p could directly bind to the 3′UTR of CXCR4 to negatively regulate CXCR4 expression.

Furthermore, we transfected OA chondrocytes with negative control mimic, miR-142-5p mimic, negative control inhibitor, and miR-142-5p inhibitor to manipulate the expression level of miR-142-5p (Figure 2(b)). We confirmed that miR-142-5p overexpression downregulated CXCR4 expression (*p*=0.0035), while CXCR4 mRNA level was significantly upregulated by the miR-142-5p inhibitor treatments (*p*=0.0076) (Figure 2(c)). Western blot showed consistent results with those of the real-time PCR assays: CXCR4 protein expression was upregulated in the miR-142-5p mimic group, while miR-142-5p inhibitor group had reduced CXCR4 expression in comparison to the controls (*p* < 0.05) (Figures 2(d) and 2(e)).

### 3.3. miR-142-5p Downregulated CXCR4 Expression and Attenuated Chondrocyte Apoptosis in SDF-1-Induced Chondrocytes

**Figure 3:**
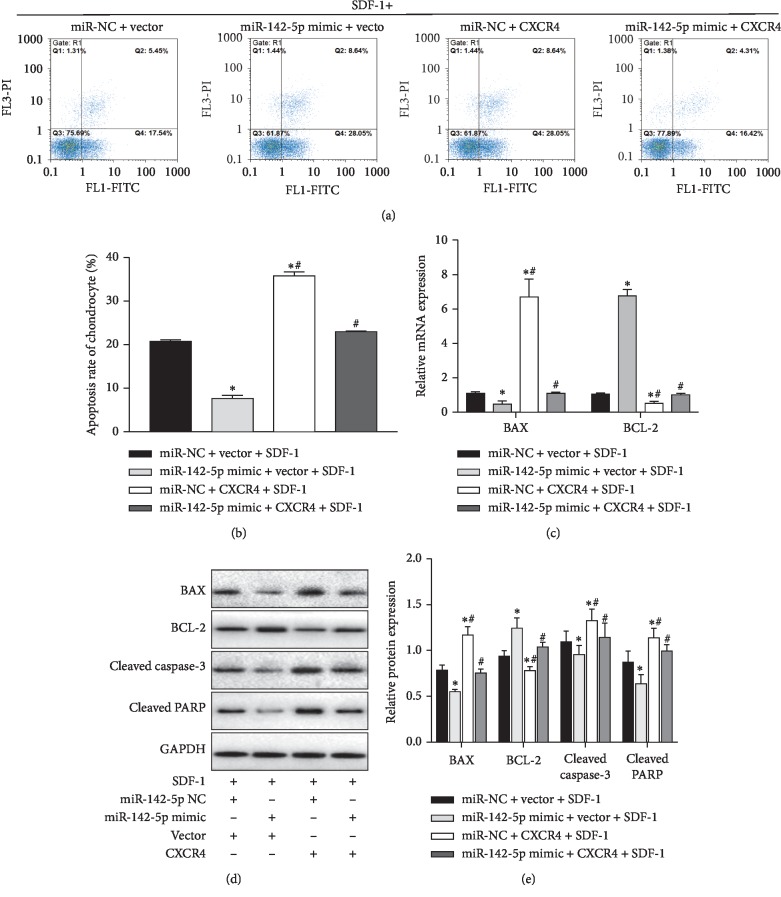
miR-142-5p overexpression attenuated SDF-1-induced chondrocyte apoptosis by targeting CXCR4. (a–e) Primary human OA chondrocytes were transfected with the indicated miRNA mimics and plasmids. At 48 h after transfection, cells were then treated with SDF-1 (100 ng/ml) for another 24 h. (a-b) Chondrocyte apoptosis was assessed by flow cytometry. The representative flow profilings of Annexin-V and PI staining are shown (a), and the rates of apoptosis in primary human OA chondrocytes were summarized (b). (c)The mRNA levels of Bax and Bcl-2 were determined with RT-qPCR. (d-e) The expression levels of cleaved caspase-3, cleaved PARP, Bax and Bcl-2 were determined by Western blot assays. The representative bands images are shown (d), and relative bands intensity was summarized (e). Data are presented as the mean ± SEM (*n* = 3). ^*∗*^*p* < 0.05; ^*∗∗*^*p* < 0.01*versus* the miR-NC + vector group; ^#^*p* < 0.05 and ^##^*p* < 0.01*versus* the miR-142-5p mimic + vector group. miRNAs, microRNAs; SDF-1, stromal cell-derived factor 1; NC, negative control; Bcl-2, B-cell lymphoma-2; cleaved PARP, cleaved poly ADP-ribose polymerase; Gapdh, glyceraldehyde‐3‐phosphate dehydrogenase.

Chondrocyte apoptosis is one of main pathology changes in pathogenesis of OA [23]. To evaluate whether miR-142-5p had an effect on regulation of SDF-induced chondrocyte apoptosis, we performed flow cytometry analysis using Annexin-V and PI staining. The OA chondrocytes were transfected with negative control miRNA mimic, miR-142-5p mimic, or the miRNA mimics together with CXCR4-expressing plasmid and were treated with 100 ng/ml SDF-1 for additional 24 hours at 24 hours after transfection. Compared with the control mimic and control vector group, miR-142-5p mimic reduced the rate of apoptosis, while CXCR4 overexpression increased the rate of apoptosis (*p* < 0.05). However, CXCR4's negative effects on chondrocyte apoptosis were weakened by miR-142-5p overexpression (*p* < 0.05), as the group with simultaneous miR-142-5p and CXCR4 expression had significantly lower apoptosis rate than the CXCR4 single overexpression group (Figures 3(a) and 3(b)). Moreover, we analyzed the expression of apoptosis-related proteins including Bax, Bcl-2, cleaved caspase-3, and cleaved PARP. As shown in Figures 3(c)–3(e), upregulation of miR-142-5p suppressed mRNA and protein expression of Bax and augmented Bcl-2 expression in SDF-1-treated chondrocytes (*p* < 0.05). In contrast, the CXCR4 overexpression increased the expressions of Bax, cleaved caspase-3, and cleaved PARP and decreased Bcl-2 expression (*p* < 0.05). In addition, CXCR4's positive effects on cell apoptosis were alleviated by miR-142-5p overexpression (*p* < 0.05). These data indicated that miR-142-5p overexpression inhibited apoptosis in SDF-1-treated chondrocytes through suppressing CXCR4.

### 3.4. miR-142-5p Counteracted Inflammation and Matrix Degradation in SDF-1-Induced Chondrocytes

**Figure 4:**
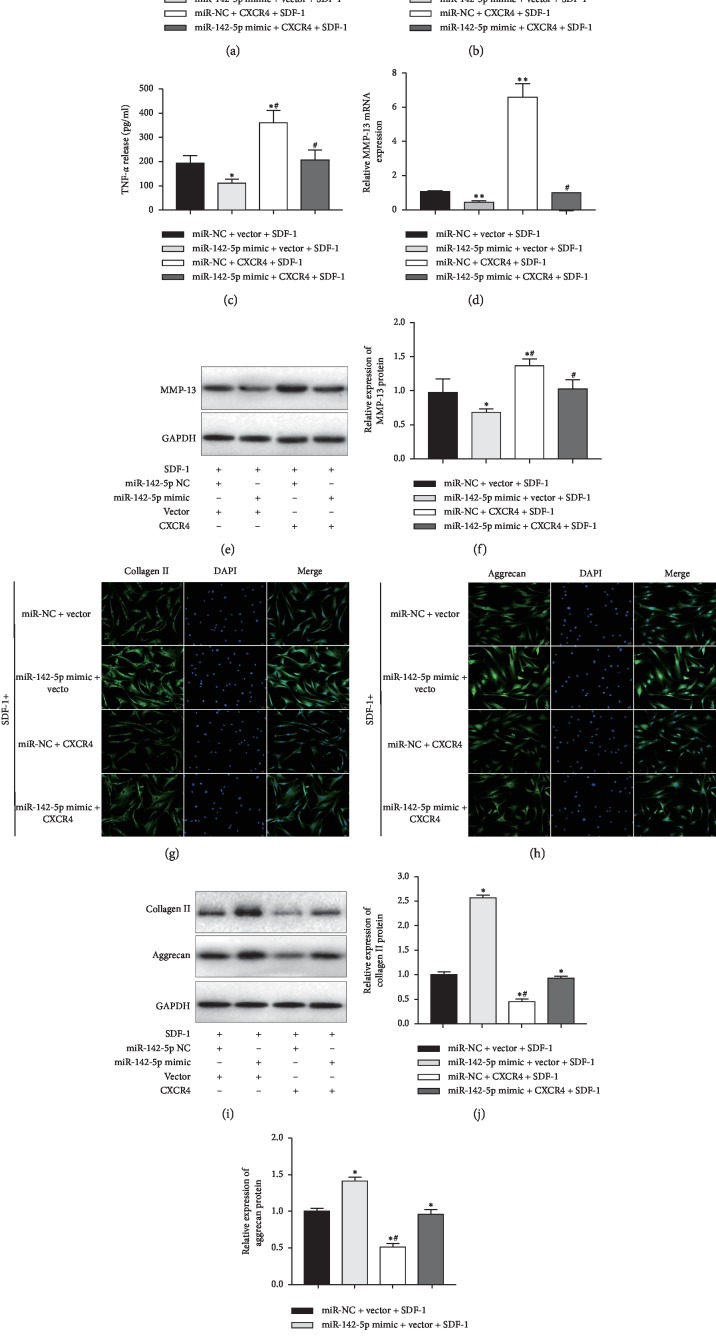
miR-142-5p counteracted inflammation and matrix degradation in SDF-1-induced chondrocytes. (a–k) Primary human OA chondrocytes were transfected with the indicated miRNA mimics and plasmids. At 48 h after transfection, cells were then treated with SDF-1(100 ng/ml) for another 24 h. The release of (a) IL-1*β*, (b) IL-10, and (c) TNF-*α* in culture supernatant was measured by ELISA kits. (d) The mRNA level of MMP-13 expression was measured by qRT-PCR. (e, f) The protein level of MMP-13 expression was measured by Western blot assays. The representative bands images are shown (e), and relative bands intensity was summarized (f). (g-h) Immunofluorescence analysis of collagen II (g) and aggrecan (h) expression in primary human OA chondrocytes after indicated transfection. Scale bar, 50 *μ*m. (i–k) The protein expressions of collagen II (j) and aggrecan (k) were determined with Western blot assays, and the representative bands images are shown (i). ^*∗*^*p* < 0.05; ^*∗∗*^*p* < 0.01*versus* the miR-NC + vector group; ^#^*p* < 0.05 and ^##^*p* < 0.01*versus* the miR-142-5p mimic + vector group. Data are represented as the mean ± SD (*n* = 3). miRNAs, microRNAs; SDF-1, stromal cell-derived factor 1; NC, negative control; IL-1*β*, interleukin-1*β*; IL-10, interleukin–10; TNF-*α*, tumor necrosis factor *α*; MMP-13, matrix metalloproteinase-13; ACAN, aggrecan; collagen II, type II collagen; Gapdh, glyceraldehyde‐3‐phosphate dehydrogenase; DAPI, 4′,6-diamidino-2-phenylindole.

To better understand the role of miR-142-5p in the pathogenesis of OA, biomarkers of inflammation and cartilage degradation, including IL-1*β*, IL-10, TNF-*α*, MMP-13, aggrecan, and collagen II [24], were measured in this study. miR-142-5p overexpression resulted in significantly decreased levels of IL-1*β* (Figure 4(a)) and TNF-*α* (Figure 4(c)) and increased level of IL-10 (Figure 4(b)), compared to control chondrocytes treated with SDF-1 (*p* < 0.05). However, overexpression of CXCR4 clearly increased the release of IL-1*β* (Figure 4(a)) and TNF-*α* (Figure 4(c)) and decreased the release of IL-10 (Figure 4(b)), which was attenuated by miR-142-5p overexpression.

In addition, miR-142-5p overexpression significantly decreased the expression of MMP-13, as evidenced by qPCR assay (Figure 4(d)) and Western blot assays (Figures 4(e) and 4(f)). It also obviously increased the expression of collagen II and aggrecan, as reveled by immunofluorescence staining (Figures 4(g) and 4(h)) and Western blot assays (Figures 4(i)–4(k)). In contrast, CXCR4 overexpression significantly upregulated MMP-13 levels and downregulated the levels of collagen II and aggrecan. Notably, the negative impacts of CXCR4 overexpression in SDF-induced chondrocytes were reversed by simultaneous upregulation of miR-142-5p, and no significant differences between the CXCR4/miR-142-5p co-overexpression group and the control group were observed. Taken together, these results confirmed that miR-142-5p can decrease inflammation and reduce ECM components (collagen II and aggrecan) loss to alleviate the degeneration of SDF-1-exposed chondrocytes.

### 3.5. The Reduction of Cartilage Injury by miR-142-5p in SDF-1-Induced Chondrocytes Was Associated with Inhibiting the MAPK Signaling Pathway

**Figure 5:**
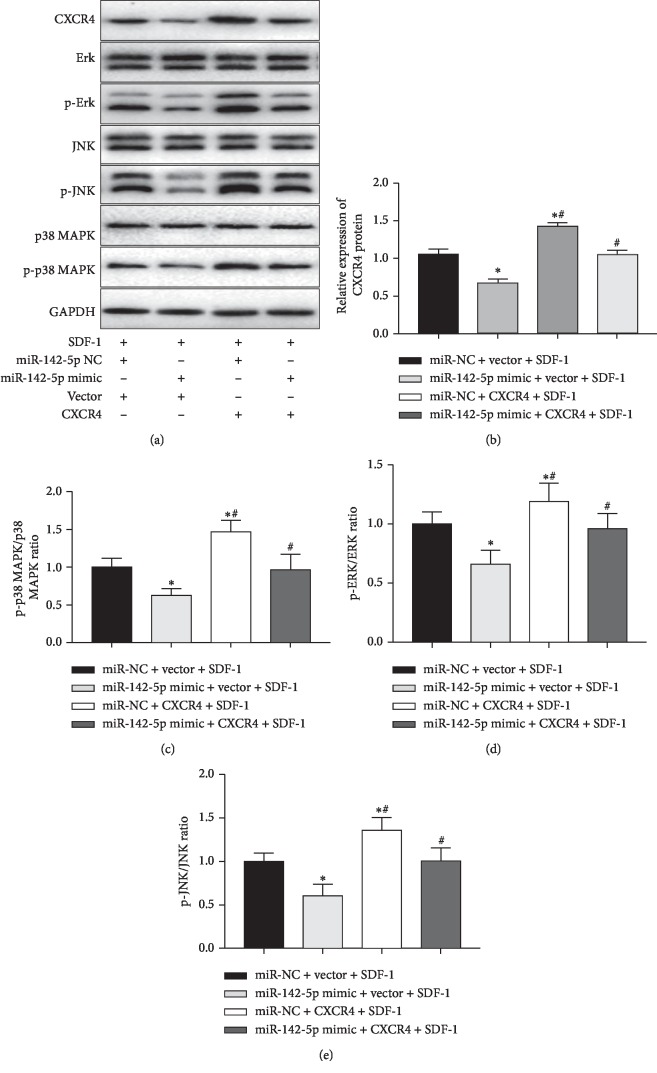
miR-142-5p regulated MAPK signaling pathways in SDF-1-induced chondrocytes. (a–e) Primary human OA chondrocytes were transfected with the indicated miRNA mimics and plasmids. At 48 h after transfection, cells were then treated with SDF-1 (100 ng/ml) for another 24 h. The protein expressions of key molecules in the MAPK (JNK, p38, ERK, p-JNK, p-p38, and p-ERK) signaling pathway were then measured by Western blot assays. Representative bands images of molecules in the MAPK (a) signaling pathways are shown. The relative bands intensity of CXCR4 (b) and the ratios of p-p38/*p*38 (c), p-ERK/ERK (d), and p-JNK/JNK (e) were summarized. Data are presented as the mean ± SEM (*n* = 3). ^*∗*^*p* < 0.05; ^*∗∗*^*p* < 0.01. miRNAs, microRNAs; SDF-1, stromal cell-derived factor 1; NC, negative control; ERK, extracellular regulated protein kinases; p-ERK, phosphorylated extracellular regulated protein kinases; JNK, c-Jun N-terminal kinase; p-JNK, phosphorylated c-Jun N-terminal kinase; Gapdh, glyceraldehyde‐3‐phosphate dehydrogenase.

To explore additional insights into the mechanisms underlying the reverse of CXCR4 overexpression mediated negative effects by miR-142-5p, we also investigated the effects of miR-142-5p on MAPK pathway, which is a typical pathway downstream of the SDF-1/CXCR4 signaling [25]. To further verify the regulatory relationship between miR-142-5p and MAPK pathway, we measured the levels of p-38, p-P38 MAPK, ERK, p-ERK, JNK, and p-JNK SDF-1-induced chondrocytes with manipulated expressions of miR-142-5p and CXCR4. As shown in the Western blot results (Figure 5(a)), overexpression of miR-142-5p conspicuously decreased the ratios of p-P-38 MAPK/P-38 MAPK (Figure 5(c)), p-ERK/ERK (Figure 5(d)), and p-JNK/JNK (Figure 5(e)), whereas overexpression of CXCR4 had opposite effects. Again, simultaneous overexpression of miR-142-5p reversed the effects of CXCR4 single overexpression on this pathway. Collectively, these data confirmed that overexpression of miR-142-5p inhibited the activation of MAPK pathway, which was associated with the ability of miR-142-5p in downregulating CXCR4 expression.

## 4. Discussion

Recently, the fact that some aberrantly expressed miRNAs play a critical role during OA pathogenesis has received increasing attention [14, 15]. In the current study, we demonstrated that miR-142-5p is significantly downregulated in cartilage tissues of patients with OA, as well as in SDF-1-treated chondrocytes *in vitro*. Through miRNA mimic transfection-mediated overexpression in OA chondrocytes, we found that miR-142-5p targets CXCR4 and plays a crucial role in OA progression, which includes increasing the expression of type II collagen and aggrecan, decreasing cell apoptosis, and reducing inflammatory factors release. Furthermore, our result revealed that upregulated miR-142-5p expression efficiently blocked MAPK signaling pathway which was triggered by the SDF-1/CXCR4 axis in OA chondrocytes.

It has been reported that SDF-1 expression in the plasma and synovial fluid of OA patients was associated with radiographic severity of knee osteoarthritis [10]. Thus, the level of SDF-1 may serve as an effective biomarker for the severity of OA [26], and chondrocytes of cartilage tissues from OA patients might be often exposed to high levels of SDF-1. Therefore, in order to mimic the osteoarthritic environment of the knee joint, we treated the isolated chondrocytes with 100 ng/ml SDF-1 in each group [27]. As a receptor of SDF-1 [7], CXCR4 has been proven to be one of the key factors that are closely involved in the pathogenesis of OA [25, 27, 28]. The binding of SDF-1 to CXCR4 induces OA cartilage degeneration [28], and it also coordinates the crosstalk between subchondral bone and articular cartilage in OA pathogenesis [29]. In addition, blocking the SDF-1/CXCR4 signaling axis has been reported to be able to inhibit cartilage degeneration in osteoarthritis by CXCR4 antagonists [13, 25, 30, 31]. In the current study, miR-142-5p was screened out through bioinformatics prediction of significantly downregulated miRNAs in cartilages from OA patients in comparison to healthy cartilages, as well as experimental validation of CXCR4-targeted and downregulated miRNAs in the chondrocytes induced with SDF-1. Similar to our results, a recent report from Jia et al. also identified miR-146-5p as a CXCR4-targeted miRNA, and its expression in OA chondrocytes was upregulated by the CXCR4 antagonist TN14003 [12]. As a CXCR4/SDF-1 axis inhibitor, miR-146a-5p also significantly attenuates SDF-1-induced cartilage degradation upon overexpression in OA chondrocytes [12]. Collectively, both our and others' results highlight the critical roles of some unappreciated miRNAs in regulating OA pathogenesis through modulating the CXCR4/SDF-1 axis.

Previous studies have shown a link between increased CXCR4 expression and impaired matrix catabolism, elevated synovial inflammation, and progressive chondrocytes number decrease during OA development [8, 11]. In line with this notion, CXCR4 inhibition can result in attenuation in OA-associated cartilage degeneration [12, 30, 31]. We identified miR-142-5p as an inhibitor of CXCR4 and a regulator of chondrocyte apoptosis. Our present study showed that upregulation of miR-142-5p resulted in clearly decreased apoptosis rate in OA chondrocytes along with significant changes in the expression levels of Bax, Bcl-2, cleaved caspase-3, and some inflammation-associated factors including IL-10, IL-1*β*, and TNF-*α*. It has been reported that miR-142-5p acts as a master regulator of osteoclast differentiation, cells proliferation, and apoptosis [32, 33]. In pancreatic cancer cells, miR-142-5p has been found to target Ras-related protein Rap-1A (RAP1A) to downregulate p-ERK1/2 and phosphate p38 mitogen-activated protein kinases (p-p38) and control cell proliferation and apoptosis [33]. Therefore, miR-142-5p can exert antiapoptosis functions through targeting other molecules other than CXCR4. We also found that the protein levels of ECM components (collagen II and aggrecan) were markedly increased after overexpression of miR-142-5p, even under the condition of CXCR4 coexpression. These results suggest that miR-42-5p alleviates chondrocyte apoptosis, inflammation factor release, and ECM deposition even in chondrocytes with active SDF-1/CXCR4 signaling, which provides rationale for clinical practice of treating OA patients with external miR-142-5p. On the other hand, since miR-142-5p is involved in many pathways such as regulating the PI3k/Akt/FoxO1 pathway via targeting PTEN in bone marrow-derived macrophages [32], more investigations are required to reveal the full mechanisms of the beneficial effects of miR-142-5p in OA chondrocytes, which might not be limited to targeting CXCR4.

Our findings also support a link between OA development and the activation of MAPK signaling, the two major OA risk factors. MAPKs, including JNK, ERK, and p38, play crucial roles in the inflammatory process and have been suggested to be extensively involved in regulating OA [34, 35]. For example, CXCR4 knockdown can inhibit SDF-1-induced angiogenesis of human umbilical vein cells, which is dependent on downregulation of the MAPK/ERK, PI3K/AKT, and the Wnt/*β*-Catenin pathways [36]. MAPK has been considered to be attractive targets for drug mediated modulation of inflammatory processes in OA [37]. Our results show that overexpression of CXCR4 activated MAPK signaling, while upregulation of miR-142-5p significantly decreased the expression of CXCR4 and inactivated MAPK signaling pathway. Since MAPK signaling pathway has been implicated in apoptosis regulation [38, 39], these findings further consolidate the antiapoptosis functions of miR-142-5p in OA chondrocytes.

## 5. Conclusions

In conclusion, through miRNA microarray and bioinformatics analyses, we identified miR-142-5p as a significantly downregulated miRNA in human OA cartilage tissues and also a CXCR4-targeted miRNA whose expression was downregulated in SDF-1-treated OA chondrocytes. Overexpression of miR-142-5p alleviated OA pathology by suppressing chondrocyte apoptosis, which was associated with decreased cartilage matrix degradation and inactivated MAPK signaling pathway possibly through direct targeting of CXCR4. Although verification of the positive effectives of *in vitro* miR-142-5p mimic administration in attenuating OA development still needs additional animal and clinical investigations, our work provides important insight into targeting miR-142-5p and the SDF-1/CXCR4 axis for developing potential therapeutic strategies for OA patients.

## Data Availability

The data used to support the findings of this study are available from the corresponding author upon request.
